# Characterization of Acetyl-CoA Carboxylases in the Basal Dinoflagellate *Amphidinium carterae*

**DOI:** 10.3390/md15060149

**Published:** 2017-05-26

**Authors:** Saddef Haq, Tsvetan R. Bachvaroff, Allen R. Place

**Affiliations:** 1 Graduate Program in Life Sciences, University of Maryland, Baltimore, MD 21201, USA; 2 Institute of Marine and Environmental Technology, University of Maryland Center for Environmental Science, Baltimore, MD 21201, USA; bachvarofft@umces.edu (T.R.B.); place@umces.edu (A.R.P.)

**Keywords:** dinoflagellate, acetyl CoA carboxylase, polyketide, fatty acids, *Amphidinium carterae*, toxin synthesis

## Abstract

Dinoflagellates make up a diverse array of fatty acids and polyketides. A necessary precursor for their synthesis is malonyl-CoA formed by carboxylating acetyl CoA using the enzyme acetyl-CoA carboxylase (ACC). To date, information on dinoflagellate ACC is limited. Through transcriptome analysis in *Amphidinium carterae,* we found three full-length homomeric type ACC sequences; no heteromeric type ACC sequences were found. We assigned the putative cellular location for these ACCs based on transit peptide predictions. Using streptavidin Western blotting along with mass spectrometry proteomics, we validated the presence of ACC proteins. Additional bands showing other biotinylated proteins were also observed. Transcript abundance for these ACCs follow the global pattern of expression for dinoflagellate mRNA messages over a diel cycle. This is one of the few descriptions at the transcriptomic and protein level of ACCs in dinoflagellates. This work provides insight into the enzymes which make the CoA precursors needed for fatty acid and toxin synthesis in dinoflagellates.

## 1. Introduction

Marine dinoflagellates, a type of phytoplankton, are primary producers in the ecosystem that are of great environmental and economic importance. Toxin producing, harmful algal bloom (HAB) species are responsible for fish mortality as well as human and animal illness worldwide [1,2,3]. Economically, dinoflagellates and algae produce important compounds such as docosahexaenoic acid (DHA) and algal biofuels [4,5]. A necessary protein involved in the production of both fatty acid (FA) products as well as polyketide (PKS) products is acetyl CoA carboxylase (ACC), which produces “activated acetate” units as malonyl CoA [6,7]. 

ACCs are natively biotinylated proteins that convert acetyl CoA (two carbons) to malonyl CoA (three carbons). Once this conversion occurs, the substrate, malonyl CoA, is committed to go through fatty acid synthase (FAS) or polyketide synthase (PKS) cycles. Within the ACC protein there are several domains. Biotin carboxylase (BC) is responsible for carboxylating the biotin cofactor, which is then transferred to the carboxyl transferase (CT) domain via the biotin carrier protein (BCCP) which serves as an attachment point for the biotin. The CT domain then carboxylates the acetyl CoA, converting it to malonyl CoA [6,8]. In addition, in the eukaryotic ACC protein there is a large middle domain, the central domain, with no currently defined function [9].

Two different ACC forms exist, a homomeric form found in most eukaryotes and a heteromeric form found in most prokaryotes. The homomeric form exists as one large polypeptide where all the subunits are on one protein with sizes typically in the 250 kD range [10]. Heteromeric subunits exist as separate proteins that work together to carboxylate acetyl CoA. Sub-unit sizes and organization are species-dependent [6,8,11].

The ACC cellular location will vary depending on the organism. In animal cells, ACCs can be found in the cytosol and mitochondria, while plant and algal ACCs are located in the cytosol and plastid [12,13]. Plastid ACC blocking compounds are used as commercially available herbicides, while mitochondrial and cytosolic versions are studied for human illnesses such as diabetes and obesity [11]. Nomenclature of ACCs also varies depending on the organism. For the purposes of our study, we will refer to plastid ACC as ACC1 and cytosolic as ACC2 in keeping with previous studies involving algal ACC [8]. 

In dinoflagellates, information about ACC and its role in fatty acid, toxin, and other secondary metabolite production is limited. *Amphidinium carterae* is a polyketide producing dinoflagellate that produces amphidinol. This linear polyketide toxin forms pores in cell membranes after complexing with sterols, resulting in cell lysis [14,15]. With characteristics such as worldwide distribution, production of amphidinol toxin, and classification as a basal dinoflagellate, *Amphidinium* is often utilized as a model dinoflagellate genus [16,17]. In our transcriptome study, we utilized *A. carterae* (CCMP 1314) to characterize and annotate ACC in dinoflagellates. 

## 2. Results

### 2.1. Transcriptome Analysis of Amphidinium carterae (CCMP 1314) ACCs

**Figure 1:**
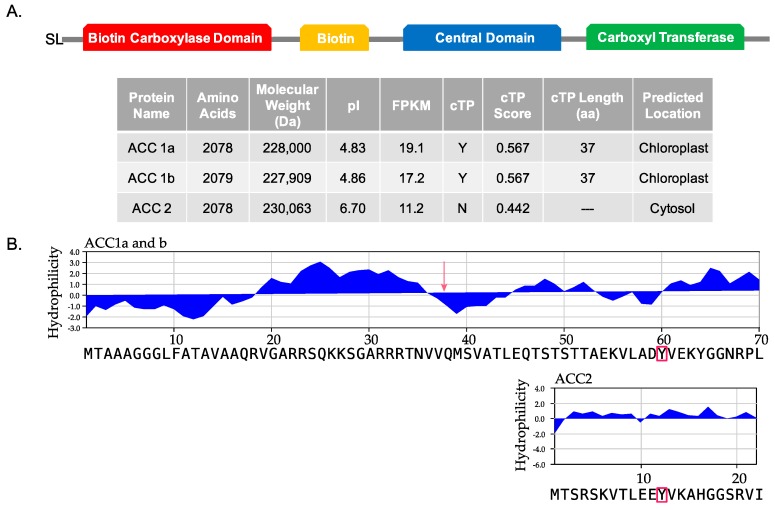
The expressed acetyl CoA carboxylases (ACCs) in *Amphidinium carterae* (CCMP 1314). (**A**) The three homomeric ACC sequences discovered through transcriptome assembly and analysis that contained the four domains of eukaryotic ACC shown above. The table shows the calculated molecular weight, pI, FPKM, cTP score, and predicted cellular location based on ChloroP. (**B**) Kyte-Doolittle hydrophilicity plot is shown for both ACC sequences. The sequence alignment is anchored by a conserved tyrosine residue shown in red boxes. A red arrow is drawn to show the predicted cleavage site of the chloroplast transit peptide.

The transcriptome analysis of *Amphidinium carterae* revealed three homomeric type ACCs containing all the domains normally present in eukaryotic homomeric ACCs in the correct order (Figure 1). We refer to these three ACC sequences as ACC1a, ACC1b, and ACC2. GenBank accession numbers are ACC1a (MF043929), ACC1b (MF043930), and ACC2 (MF043931). The ACC1a assembled transcript began with a 144 base 5′ untranslated region (UTR), a 6327 base open reading frame (ORF) and a 210 base 3′ UTR. ACC1b contained a 144 base 5′ UTR, a 6240 base ORF and a 26 base 3′ UTR. The ACC2 transcript began with eight bases of spliced leader (SL) sequence, followed by a 67 base 5′ UTR, a 6237 base ORF, and ended with a 133 base 3′ UTR. Based on the pairwise alignment of ACC1 and ACC2 using BlastP, the two proteins were 47% identical over 2047 amino acids. The two subtypes, ACC1a and ACC1b, were 98% identical over 2044 amino acids and 98% identical over 6374 nucleotides (Figure 1A).

Based on the Center for Biological Sequence Analysis (CBS) ChloroP prediction software, ACC1a and 1b contained a chloroplast transit peptide (cTP), with a cTP score of 0.567, while ACC2, with a cTP score of 0.442, did not [18,19,20]. The cTP length was predicted to be 37 amino acids for both ACC1a and 1b (Figure 1A). When the three ACC sequences were aligned, ACC1a and 1b started 48 residues before ACC2. This offset was demonstrated by the alignment of a conserved tyrosine at residue 60 of ACC1a and 1b, which corresponded to residue 12 of ACC2. For the initial 48 residues, Kyte-Doolittle hydrophilicity plots revealed a pattern of 18 hydrophobic residues immediately preceding the predicted cTP, followed by a seven amino acid hydrophilic region in ACC1a and 1b. ACC2 did not have a similar pattern of residues (Figure 1B) [21].

### 2.2. Quantification of ACC Transcript Levels in Amphidinium carterae

**Figure 2:**
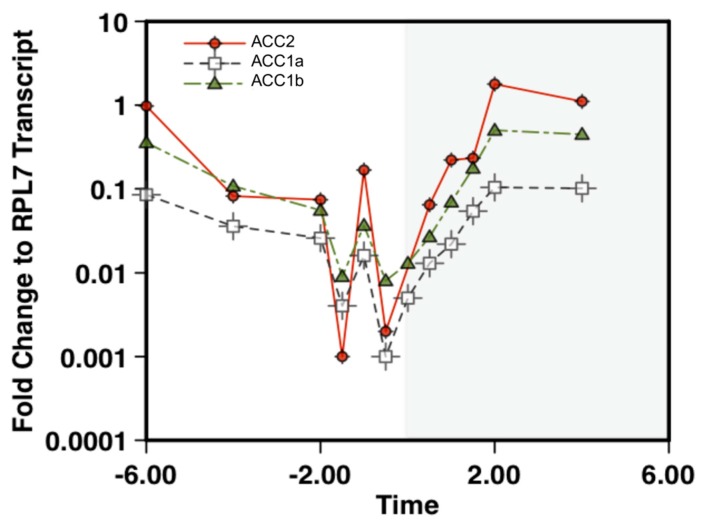
Quantification of ACC mRNA in *Amphidinium carterae*. Cells were collected at one hour increments 6 h before and after the light:dark transition. The white background shows the light period (T − Hour) while the shaded region shows the dark period (T + Hour) with T0 representing when the lights are turned off. The mRNA was quantified using quantitative reverse transcriptase PCR to observe the fold change relative to RPL7 as a native reference gene. The apparent peak at −1 h is due to a greater drop in RPL7 transcript abundance than observed with the ACCs.

ACC mRNA quantification was consistent with the global transcript patterns in dinoflagellates over a diel light cycle. Previously, in *A. carterae*, global changes in mRNA abundance were observed near the light:dark transition [22,23]. The ACC transcripts closely followed this pattern with decreasing abundance towards the end of the light period, followed by recovery in the dark period (Figure 2). Ribosomal protein L7 (RPL7) was used as a native reference gene. Cycle thresholds for ACC were normalized to RPL7 values, and all samples were run in triplicate. The transcript abundance was generally ACC2 > ACC1b > ACC1a at most time points, albeit with some reordering during the very low abundance measures around the light:dark transition. 

### 2.3. Profile of Natively Biotinylated Proteins in Amphidinium carterae 

**Figure 3:**
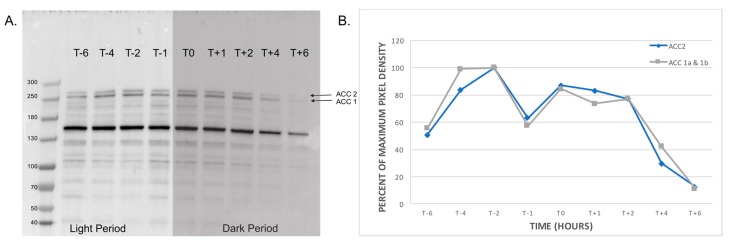
Streptavidin Western blotting of *Amphidinium carterae*. (**A**) Natively biotinylated proteins were surveyed over a 12-h period using Western blotting with streptavidin horseradish peroxidase conjugate at 300,000 cell equivalents per time point in *A. carterae*. T0 represents the time point when the lights were turned off, with hours before and after represented as T − hour and T + hour, respectively. (**B**) Densitometry pixel densities were normalized to the maximum density peak and plotted against time for ACC1a, 1b and 2.

Streptavidin-HRP Western blot over the diel cycle shows the natively biotinylated proteins in *A. carterae* ranging from 40 to 280 kD. Samples were collected 6 h before and after the light:dark transition, and 300,000 cell equivalents per lane were run for each time point. Two bands are observed at 272 kD and 258 kD (Figure 3), which are consistent with, but larger than the estimated sizes of the different ACCs observed in the transcriptome analysis (Figure 1). A prominent band is also observed at 160 kD; we hypothesize this to be pyruvate carboxylase, but further experiments are required to verify this assignment. Densitometry results that were normalized to the maximum pixel density show a slight decrease in protein abundance at 6 h before and 6 h after the light:dark transition. The highest protein abundance was observed 2–4 h prior to the dark period (Figure 3).

### 2.4. Peptide Coverage of ACCs in Amphidinium carterae

**Figure 4:**
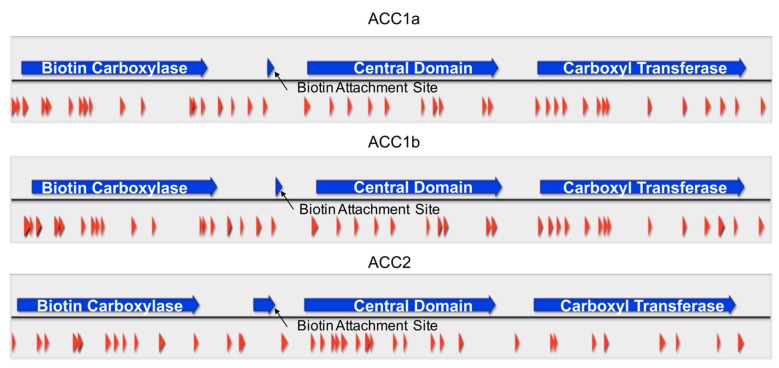
Mass spectrometry proteomics of ACCs in *Amphidinium carterae*. Peptide sequences were determined using mass spectrometry proteomics techniques. Peptide coverage is represented with red arrows, while blue arrows indicate different ACC domains. Black arrows denote the biotin attachment site in each sequence.

A shotgun mass spectrometry experiment was conducted using high molecular weight protein bands (approximately 220, 160 kD) that were separated on sodium dodecyl sulfate polyacrylamide gel electrophoresis (SDS-PAGE). The gel bands were then digested using trypsin, producing peptide fragments. Many of these peptide fragments could be mapped back to the three ACCs in *A. carterae* and covered all the domains in these proteins. The highest number of peptide fragments to map back to ACC were present in the 220 kD gel band. Peptide coverage of 20.3%, 20.4%, and 18.5% are observed for ACC1a, ACC1b, and ACC2, respectively (Figure 4). The high similarity between ACC1a and 1b mean that some peptides mapped to both predicted proteins. 

## 3. Discussion

ACCs are the rate-limiting commitment step in fatty acid and polyketide synthesis [7,24]. Due to their involvement in essential processes, these proteins have been heavily studied in many organisms. However, little is known about ACCs in dinoflagellates, which are presumably involved in fatty acid and toxin synthesis. In this study, we describe ACCs present in a basal toxin-producing dinoflagellate *Amphidinium carterae*. 

We began our investigation by searching our transcriptome database for ACCs in *A. carterae* [25]. This analysis uncovered three sequences that contained all the domains ordinarily found in eukaryotic ACCs; no heteromeric type ACCs were found. A biotin carboxylase domain, a biotin attachment site, a central domain, and carboxyl transferase domain were all present (Figure 1). Finding these domains on one polypeptide in addition to the inclusion of the central domain, a non-catalytic domain specific to eukaryotic ACCs, was consistent with a homomeric type ACC [9]. 

We predict that the two types of ACCs compartmentalize to different cellular locations where ACC1a and 1b are targeted to the plastid, while ACC2 is cytosolic. In addition to phylogenetic analysis, we observe in the ACC1 sequences a chloroplast transit peptide (cTP) with a hydrophobicity pattern consistent with a signaling peptide (Figure 1) [21,26]. This is similar to other plastid-containing organisms that are phylogenetically similar, such as *Toxoplasma gondii*, which has two different homomeric ACCs, one found in the plastid while the other is found in the cytosol [27]. This varies from plants such as *Arabidopsis thaliana*, which contain two cytosolic versions of ACC [8,28].

This prediction is validated by high cTP scores for ACC1a and 1b when analyzed by the ChloroP predictive software (Figure 1). ChloroP outputs cTP scores between 0.4 and 0.6, where scores above 0.5 are predicted to contain a cTP. These sequences have a cTP score that exceeds the 0.55 threshold, which is considered a strong prediction for the presence of a cTP [18]. In addition, when these sequences are aligned to a tyrosine residue that is conserved across multiple dinoflagellate and algal species, ACC1a and 1b have an additional 48 amino acids at the N-terminus of the protein sequence (Figure 1) [21]. This 48 amino acid sequence in ACC1a and 1b contains a group of hydrophobic amino acids immediately preceding the predicted site of the transit peptide, a pattern that has been observed in plastid-targeted proteins in other dinoflagellates [26]. ACC2 did not have a predicted transit peptide, nor was there a pattern of hydrophobic residues at the N-terminus of the sequence. Taken together, we reason that ACC1a and 1b are located in the plastid, while ACC2 is located in the cytosol of *A. carterae*. 

We further validated the presence of ACCs in *A. carterae* by Western blotting and mass spectrometry proteomics to verify the presence of these proteins, as dinoflagellates exhibit high levels of translational control [22,23]. Using streptavidin-HRP Western blotting on axenic *A. carterae*, we obtained a profile of all natively biotinylated proteins over a diel cycle (Figure 3). Two bands observed at 272 kD and 258 kD were putatively assigned as our ACCs of interest, ACC2 and ACC1, respectively. We also observed a slight decrease in protein abundance at the time points furthest from the end of the light:dark transition at −6 and +6 h (Figure 3). 

Mass spectrometry proteomics results show extensive peptide coverage of all three ACCs observed in *A. carterae* (Figure 4). Due to the high peptide coverage (Figure 4) and high expression level (fragments per kilobase per million mapped reads) (Figure 1), these proteins are abundantly expressed and translated in the cell [25]. These proteins are present in large amounts likely because they catalyze a limiting step in essential fatty acid synthesis and the production of secondary metabolites through polyketide synthases [29]. These proteins now represent an attractive target for further dissection of the PKS/FAS pathways in dinoflagellates.

## 4. Conclusions

To date, few descriptions of acetyl CoA carboxylases in dinoflagellates exist [30]. Our current study shows the presence of two types of homomeric type ACCs in a basal dinoflagellate, *A. carterae*. We plan to further characterize these ACCs with phylogenetic comparisons to other species as well as functional studies to show their involvement in fatty acid and polyketide production. 

## 5. Materials and Methods

### 5.1. Sequence Acquisition and Searching

The transcriptome used in this study was assembled from the 100 base paired end reads in the short read archive SRX722011 at NCBI, assembled de novo with Trinity [31]. The assembled data was compared to the NCBI reference sequence protein database by BLASTx with e-value cut off of 1 × e^−10^ and the results were compiled into a relational database. Putative ACC sequences were extracted based on the sequence descriptions in the BLAST database and annotated using conserved domain search tool as a default of online (interactive) BLAST against the NCBI conserved domain database (CDD). Iterative searching using individual conserved domains was then conducted; each sequence extracted from *A. carterae* was compared to the CDD database using interactive BLAST to NCBI, tabulated, and then used to extract similar sequences from *A. carterae* (using BLASTx with evalue cutoff of 1 × e^−10^). Any novel sequences were extracted, translated, and compared to the CDD, and added to the query set. When no new sequences with either the biotin carboxyl carrier protein (cd06850), ACC synthase central domain (pfam08326), or the carboxytransferase (pfam01039) conserved domains were found, the search was terminated. Only sequences with the domain architecture shown in Figure 1 were considered genuine ACC synthases. No single subunit sequences corresponding to heteromeric ACC synthases, and with matches to individual domains, were found. 

### 5.2. ChloroP Analysis of Sequences

Amino acid sequences for ACCs in *A. carterae* were analyzed using the CBS ChloroP predictive software http://www.cbs.dtu.dk/services/ChloroP/. Sequences were pasted into the input window in FASTA format and jobs were submitted using detailed output analysis. cTP scores were evaluated using cut-offs published by the CBS group [18].

### 5.3. Kyte-Doolittle Hydrophilicity Analysis

Kyte-Doolittle analysis was conducted on the first 100 amino acids of ACC protein sequences in *A. carterae* using Kyte-Doolittle hydrophilicity analysis in the protein analysis toolbox feature of MacVector analysis software (Version 15.1.5, MacVector Inc., Apex, NC, USA). 

### 5.4. Cell Culture and Harvest for Diel Quantification

*Amphidinium carterae* (CCMP 1314) was grown in enriched seawater artificial water (ESAW) medium modified to contain 10 mM HEPES in a 20-L multiport polycarbonate carboy [32]. The cultures were maintained under constant light at 150 μm·cm^−2^·s^−1^ with a 14:10 light:dark schedule and bubbling of air infused with CO_2_ controlled by an American Marine (Ridgefield, CT) pH controller set to turn on above pH 8.2 and turn off at pH 7.6. Two liters of medium was added to an actively growing culture on Monday, Wednesday, and Friday, until a cell density of 172,000 cells/mL was reached on the morning of the experiment. Then, 250 mL of culture was aliquoted into 26 × 75 cm^2^ polystyrene culture flasks from Corning (Corning, NY, USA) and placed along the light bank to achieve equivalent light exposure as the stock culture in two rows of 13, with duplicates in an opposing direction. Each duplicate pair was taken for harvest according to the following schedule relative to the transition from light to dark in hours: −6.0, −4.0, −2.0, −1.5, −1.0, −0.5, 0, 0.5, 1.0, 1.5, 2.0, 4.0, 6.0. The samples were each split into 50 mL and 200 mL aliquots. Each aliquot was centrifuged at 1000× *g* for 10 min at 20 °C. The 200 mL aliquot was frozen at −80 °C and the 50 mL aliquot was resuspended in 1 mL of TRI Reagent^®^ (Sigma-Aldrich, Saint Louis, MO, USA).

### 5.5. RNA Extraction and qPCR Analysis

*Amphidinium carterae* samples from each time point were extracted using TRI Reagent^®^ according to the manufacturer’s protocol using 1 mL of reagent. RNA was quantified on a Qubit™ Fluorometric Quantitation system (Thermo Fisher Scientific, Waltham, MA, USA). RNA was reverse transcribed using SuperScript™ II reverse transcriptase (Invitrogen by Life Technologies) with Random Primers (Invitrogen, Carlsbad, CA, USA) as per the manufacturer’s protocol. The cDNA was used as a template for quantitative real-time PCR using an Applied Biosystems (Life Technologies, Carlsbad, CA, USA) Fast 7500 thermal cycler in triplicate, with the following reaction setup and primers listed in Table 1: 6 μL diethyl pyrocarbonate (DEPC) treated water, 2 μL of combined forward and reverse primers at 5 μm each, 10 μL of iTaq™ 2X master mix containing SYBR™ green and ROX (Bio-Rad, Hercules, CA, USA), and 2 μL of template cDNA at 10 ng/μL. Thermal cycling conditions consisted of 40 cycles of an initial denaturation at 95 °C for 2 min followed by 40 cycles of denaturation at 95 °C for 15 s, and annealing and fluorescent data collection at 60 °C for 1 min. Cycle thresholds and baselines were determined manually and were averaged and compared across time points. Cycle thresholds of triplicate ACC samples were averaged and those values were normalized to C_T_ values of RPL7. 

### 5.6. Cell Culture and Harvest for SDS-PAGE and Mass Spectrometry

*Amphidinium carterae* was cultured as described above. Cultures were grown to a high cell density (300,000 cells/mL or higher) as measured with a Coulter counter. Cells were collected at midday time points. Aliquots (50 mL) of culture were poured into a 50-mL conical tube and centrifuged at 350× *g* for 10 min at room temperature. The supernatant was poured off and the tube containing the pellet was placed in a bath containing ethanol and dry ice until frozen. The samples were then stored at −80 °C until analysis. 

### 5.7. Western Blotting

Cell pellets of *A. carterae* were resuspended in 1 mL of 1X SDS sample buffer (50 mM Tris-Cl pH 6.8, 2% SDS, 0.1% bromophenol blue, 10% glycerol, 100 mM dithiothreitol), incubated for 5 min at 95 °C, and centrifuged at 10,000× *g* for 5 min. Samples were loaded onto gel at 300,000 cell equivalents per lane and separated on Novex™ NuPAGE 3–8% tris-acetate gels at 150 V (constant) for 1 h. Proteins were transferred onto a polyvinylidene difluoride (PVDF) membrane using the high molecular weight program on the Trans Blot^®^ Turbo™ Transfer System (Bio-Rad) for 14 min. The membrane was rinsed with deionized water and blocked with 1X iBind™ solution (Life Technologies) for 1 h at room temperature. The membrane was incubated with a streptavidin-HRP conjugate using the iBind™ Western Device (Life Technologies) as per the manufacturer’s protocol at a concentration of 1:1000. Blot was incubated for 5 min in Clarity ECL Substrate (Bio-Rad) and imaged on the ChemiDoc Touch Imaging System by Bio-Rad. 

### 5.8. Preparation of Proteins for Mass Spectrometry

The *A. carterae* cell pellet was resuspended in 1X SDS sample buffer (50 mM Tris-Cl pH 6.8, 2% SDS, 0.1% bromophenol blue, 10% glycerol, 100 mM dithiothreitol), incubated for 5 min at 95 °C, and centrifuged at 10,000× *g* for 5 min. 500,000 cell equivalents per lane were loaded and run on Novex™ NuPAGE 3–8% tris-acetate gels as per the manufacturer’s protocol. Gel bands corresponding to the location of the presumptive proteins were excised with a sterile scalpel. Samples were processed using the In-Gel Tryptic Digestion Kit (Thermo Fisher Scientific) as per the manufacturer’s protocol. Gel bands were destained twice with 200 μL destaining solution (~25 mM ammonium bicarbonate in 50% acetonitrile) and incubated at 37 °C with shaking for 30 min. Samples were reduced by incubation at 60 °C for 10 min in 50 mM TCEP (tris(2-carboxyethyl)phosphine) in 25 mM ammonium bicarbonate buffer. Free sulfhydryl groups were alkylated by incubation in 100 mM iodoacetamide at room temperature for 1 h in the dark. Gel pieces were shrunk in 100% acetonitrile. The acetonitrile was removed and the gel band was allowed to air dry for 10 min. The dried gel pieces were treated with 100 ng trypsin, in a 25 mM ammonium bicarbonate buffer, and incubated overnight at 30 °C with shaking. The supernatant was removed, placed in a clean tube, and then dried completely in a SpeedVac and stored at −20 °C until analysis. 

### 5.9. Mass Spectrometry Analysis and Data Processing

Peptide samples were analyzed by electrospray ionization on a tandem Orbitrap Elite mass spectrometer (Thermo Scientific Inc.). Nanoflow HPLC was performed by using a Waters NanoAcquity HPLC system (Waters Corporation, Milford, MA, USA). Peptides were trapped on a fused-silica pre-column (100 μm i.d. 365 μm o.d.) packed with 2 cm of 5 μm (200 Å) Magic C18 reverse-phase particles (Michrom Bioresources, Inc., Auburn, CA, USA). Subsequent peptide separation was conducted on a 75 μm i.d. × 180 mm long analytical column constructed in-house and packed with 5 μm (100 Å) Magic C18 particles, using a Sutter Instruments P-2000 CO_2_ laser puller (Sutter Instrument Company, Novato, CA, USA). The mobile phase A was 0.1% formic acid in water and the mobile phase B was 0.1% formic acid in acetonitrile. Peptide separation was performed at 250 nL/min in a 95-min run, in which mobile phase B started at 5%, increased to 35% at 60 min, 80% at 65 min, followed by a 5-min wash at 80% and a 25-min re-equilibration at 5%. Ion source conditions were optimized by using the tuning and calibration solution recommended by the instrument provider. Data were acquired by using Xcalibur (version 2.8, Thermo Scientific Inc.). MS data was collected by top-15 data-dependent acquisition. Full MS scan of the range of 350–2000 *m*/*z* was performed with 60 K resolution in the Orbitrap followed by collision-induced dissociation (CID) fragmentation of precursors in an ion trap at the normalized collision energy of 35. The MS/MS spectra of product ions were collected in rapid scan mode.

Acquired tandem mass spectra were searched for sequence matches against the UniprotKB database as well as a six-frame translation of the transcriptome data using COMET. The following modifications were set as search parameters: Peptide mass tolerance at 10 ppm, trypsin digestion cleavage after K or R (except when followed by P), one allowed missed cleavage site, carboxymethylated cysteines (static modification), and oxidized methionines (variable modification/differential search option). PeptideProphet and ProteinProphet, which compute a probability likelihood of each identification being correct, were used for statistical analysis of the search results. PeptideProphet probability ≥ 0.9 and ProteinProphet probability ≥ 0.95 were used for positive identification at an error rate of less than 1%. Only proteins identified by more than one unique peptide sequence were included in the study. 

## Figures and Tables

**Table 1:** Primer sequences for mRNA quantification experiments.

Primer	Sequence	Product Size (bp)	Tm (°C)
ACC1a_F	CTTGGCGTTCTTGATGGAGT	701	55.2
ACC1a_R	TCATGATCTTTGCGAACTGG	701	53.0
ACC1b_F	TCCAAATAGGATGCCACCTC	680	54.4
ACC1b_R	TTGCCTGTCATGATCTTTGC	680	53.5
ACC2_F	ACAAACTTGTGGGGCAGTTC	701	56.0
ACC2_R	TCCCTGCAATACTTCCCATC	701	54.4
